# Research on In-Plane Deformation Performance of Rotating Honeycomb Structures

**DOI:** 10.3390/ma16175993

**Published:** 2023-08-31

**Authors:** Yongzhong Zhang, Yunhai Ma, Xue Guo, Qingyang Wang

**Affiliations:** 1 School of Biological and Agricultural Engineering, Jilin University, Changchun 130022, China; swordbeijing@163.com (Y.Z.); guoxue21@jlu.edu.cn (X.G.); qywang22@jlu.edu.cn (Q.W.); 2 Intelligent Electronic Manufacturing Research Center, Beijing City University, Beijing 101309, China

**Keywords:** Bionics Engineering, honeycomb structure, energy absorption, numerical simulation

## Abstract

Most natural materials have rotational and hierarchical properties, so they can show excellent mechanical properties such as shear resistance and impact resistance. In order to further improve the energy absorption characteristics of vibration absorbing structures, a new type of honeycomb structure with integral rotation and group rotation is designed and characterized. The effects of the geometrical parameters of rotation Angle on the impact deformation mode, stress response curve and energy absorption characteristics of the honeycomb structure are studied through numerical simulation and experimental design. The results show that the overall honeycomb performance of 15° is better than that of 0°, the specific energy absorption is the results show that the overall honeycomb performance of 15° is better than that of 0°, the specific energy absorption is increased by 6%, the bearing capacity is increased by 320 N, and the crushing force efficiency is increased by 2%. Compared with the whole cell and the group cell, the specific absorption energy increased by 35%, 73% and 71%. The results of this paper provide a new insight into the impact performance of monolithic and grouped rotating honeycomb structures, which is helpful for the results of this paper provide a new insight into the impact performance of monolithic and grouped rotating honeycomb structures, which is helpful for the optimization of crashworthiness structural design.

## 1. Introduction

As a typical periodic porous energy-absorbing structure, the honeycomb structure is mainly characterised by dynamic instability under impact loads, with the honeycomb structure producing violent plastic deformation and absorbing most of the impact energy, with excellent deformation load-bearing capacity, energy absorption, and highly desirable impact resistance, and is widely used in many engineering applications, it is widely used in bridge [1], aerospace [2,3], automobile [4,5] and other fields. With the popularisation of the concept of lightweighting and the development of technology, the use of honeycomb structures is becoming more and more common. The increasing demand for energy absorption capacity and compression resistance has led to innovative designs of shape forms and arrangements of honeycomb structures that have received much attention from scholars at home and abroad. Studies on periodic honeycomb structures with different shapes or arrangement distributions have shown that innovations in the structure of individual honeycomb cells can improve the overall mechanical properties of honeycomb structures. Hou Xiuhui et al. [6] analysed the deformation patterns of multi-concave honeycomb structures under different velocity impact loads. Zhenfeng Shen et al. [7] designed a new model of the in-concave annular honeycomb structure with negative Poisson’s ratio based on the theory of mechanical metamaterials based on the in-concave hexagonal honeycomb structure and investigated its impact response characteristics. Khan et al. [8] proposed a reentrant honeycomb structure model to improve the elastic properties of the structure by adding cell ribs to the in-concave honeycomb structure. Yi-Xian Du et al. [9] used topology optimisation for the innovative design of a honeycomb-like sandwich configuration to obtain a structure with better load-bearing performance in the coplanar direction. Liang et al. [10] added smaller hexagons at the centre and apex of the honeycomb structure to achieve a multi-stage self-similar honeycomb structure with energy absorption. Xu et al. [11] performed in-plane dynamic response and multi-objective optimisation of a sinusoidal curve with negative Poisson’s ratio honeycomb structure. The development of 3D printing technology has made it possible to fabricate multi-cell structures with fine geometric features. Wu et al. [12] proposed various types of 2D and 3D chiral mechanical metamaterials using the nodal rotation and ligament deformation properties of chiral elements for devices such as deformable airfoils with chiral core configurations; Zhang et al. [13] proposed a new anti-chiral structure with an arc-shaped ligament by replacing the straight ligament with a circular arc-shaped ligament, with an 8-fold increase in Young’s modulus. 8 times higher Young’s modulus and 4 times higher specific energy absorption. Wang et al. [14] proposed a new layered lattice design method replacing straight beams with a string of higher-order circular beams. Hamidreza Eipakchi and Farid Mahboubi Nasrekani [15,16] discussed the influence of negative Posson ratio honeycomb structure and length-diameter ratio on buckling load, and changed the mechanical properties of honeycomb structure by changing its geometric parameters. Hossein Taghipoor and Mohammad Damghani Nouri [17] have studied sandwich structures with expanded metal sheets, indicated the cell orientation was a critical parameter affecting the failure mode and energy absorption capability, the porous and lightweight honeycomb lattice structures mentioned above have been used in many applications, covering a wide range of fields such as automotive, defence, aerospace, and armour protection.

Structural bionics is inspired by the microscopic, fine, and macroscopic tissues with excellent mechanical properties of a wide variety of organisms in nature and applies them to structural design through imitation, reproduction, and optimisation to improve structural performance. In nature, organisms have evolved many structures with excellent impact resistance in order to adapt to the harsh external environment and resist invasion by natural enemies. Liu et al. [18] designed the arrangement of lattice structures based on the mechanical properties of the top, middle and abdominal structures of beetles; Yu established a negative Poisson’s ratio structure mimicking the peanut shell structure by structural bionics [19]; Wu Fei [20] designed a lotus root structure, a lotus horsetail structure and a lotus honeycomb structure inspired by the lotus structure and performed axial radial and triple bending crushing experiments; The Brigantine structure with excellent impact resistance was discovered in the structures of fish scales [21] and arthropod exoskeletons (mantis shrimp [22], lobster [23], beetle sheath wings [24], etc.), which is a helical structure consisting of planar rotating stacks of geotinous fibers, protein matrix and calcium carbonate, etc. Yu et al. [25] designed single-rotation and multi-rotation honeycomb structures based on the microstructure of grass stem cross-sections; Jiang et al. [26] designed non-linear rotational angle spiral laminates with improved impact resistance based on the microscopic spiral structure inspired by biological microstructures, and used 3D printing technology to prepare eight types of spiral angle tubular structures, and conducted compression tests and finite element simulations to elucidate their deformation structures and energy absorption principles; Liu et al. [27] found that spiral specimens outperformed unidirectional specimens in terms of impact duration, peak impact force and energy absorption in the concrete domain. D. Ginzburg et al. [28] found that spiral laminates enhanced the damage tolerance and impact energy absorption of laminated materials with minimal fibre damage. In this paper, we design a rotating honeycomb structure based on a structural bionic approach, verify the validity of the model using finite element analysis and experimental tests, and investigate the effect of angular parameters on the deformation pattern, mechanical response, and energy absorption characteristics of the honeycomb structure under the action of external loads to provide a reference for the design of energy-absorbing protective structure configurations and their application in engineering.

## 2. Materials and Methods

### 2.1. Model Design

**Figure 1:**
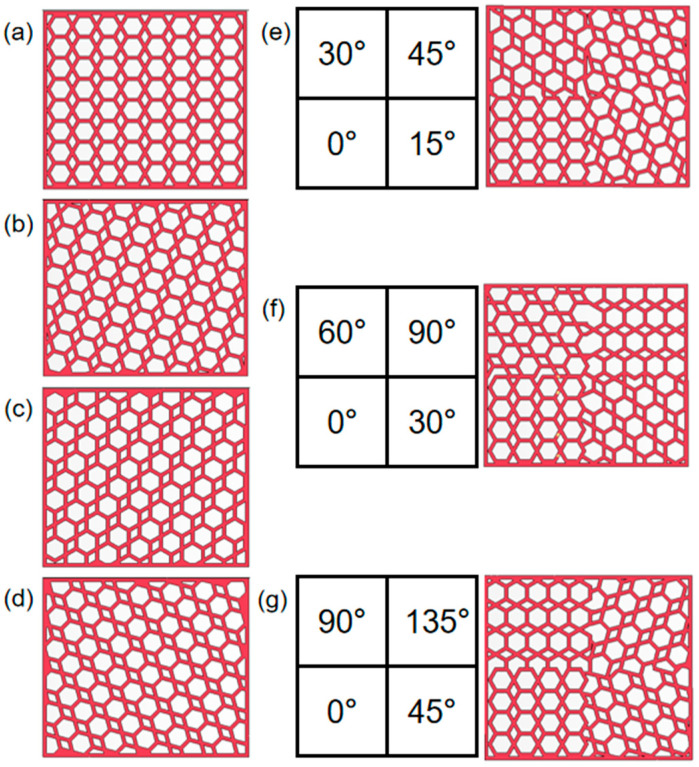
Schematic diagram of a rotating honeycomb: (**a**) 0° honeycomb, (**b**) 15° integral honeycomb, (**c**) 30° integral honeycomb, (**d**) 45° integral honeycomb, (**e**) 15° combined honeycomb, (**f**) 30° combined honeycomb, (**g**) 45° combined honeycomb.

In this paper, based on the design concept of bionic structure, combined with the deformation characteristics of honeycomb structure, in order to improve the energy absorption characteristics and impact load consistency of the ortho-hexagonal honeycomb structure, the hexagonal honeycomb structure is rotated as a whole to form a new inclined side cell wall, and four different angles of honeycomb model are proposed. Figure 1 shows the schematic diagram of the honeycomb structure. The configuration of the rotating honeycomb structure is inspired by the rotation Angle in the honeycomb structure and spiral structure. The hexagonal honeycomb structure is rotated 15°, 30° and 45° successively, ensuring that the length L = 60 mm and the height H = 50 mm of the whole structure are unchanged. The Angle of each cell wall is changed in the arrangement, and the rotation of the structure will change the force mode and the way of force transfer during the in-plane compression of the inner hexagonal cell. Based on the combined concept, the overall test frame model was densely arranged using an 8 × 8 matrix, and the matrix frame length × width × height was 60 mm × 30 mm × 50 mm, as shown in Figure 1. The effect of the cellular microstructure parameters on the internal impact performance was investigated.

**Figure 2:**
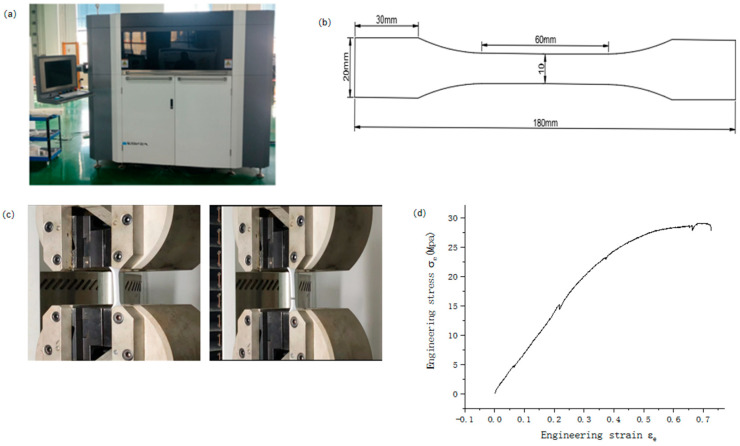
(**a**) Tensile displacement curve of the 3D printing device FS403P (**b**) dog bone sample (**c**) tensile experiment (**d**) nylon material.

According to the specific requirements of this study, this paper uses nylon material for the rapid preparation of rotating honeycomb specimens (Figure 2a) on the 3D printing equipment FS403P, which has the advantages of wear resistance, heat deformation resistance, and chemical corrosion resistance. In order to obtain the mechanical properties of the nylon material used in 3D printing, three standard tensile specimen samples with dimensions referenced to the ISO 527-2:1993 standard were produced and cut out and uniaxial tensile experiments were carried out using a universal testing machine DDL100, the average value of the three tensile samples was taken to obtain the tensile stress-strain curve as shown in Figure 2b. The detailed parameters of the nylon material are as follows: density 1100 kg/m^3^, modulus of elasticity 520 MPa, Poisson’s ratio 0.8, yield stress 23.36 MPa and tensile stress 43.00 MP.

Prior to the compression test, the honeycomb was placed in a dry and ventilated area to remove any moisture absorbed by the honeycomb and to homogenise it. The dimensions were then measured using vernier calipers and weighed using an electronic balance, and Table 1 below shows the measured dimensions and mass parameters of each rotating honeycomb.

### 2.2. Finite Element Simulation Modeling Methods

**Figure 3:**
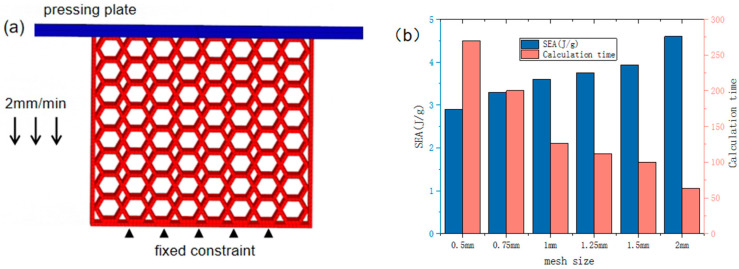
(**a**) Rotating honeycomb finite element model (**b**) Mesh convergence analysis.

Figure 3a shows a finite element model of a hexagonal rotating honeycomb structure under in-plane impact loading. The overall model consists of an upper steel plate and a rotating honeycomb structure with a fixed end at the bottom. The nonlinear display dynamic finite element software LS-DYNA is used to systematically simulate the dynamic characteristics of the integral rotation and group rotation finite element models. In order to better simulate the compression deformation of the structure, the elastoplastic material model MAT_24 and the stiffness material model MAT_20 in LS-DYNA were used to model the honeycomb structure and the rigid wall respectively. The cellular structure is modeled by hexahedral lattice elements. The automatic single-sided contact algorithm was used to prevent the penetration of the cell wall, and automatic face-to-face contact was used to simulate the contact behaviour between all cell walls and the rigid plate, with the friction factor set to 0.2 for all contacts. Boundary conditions are set for the rigid wall and the fixed end so that the rigid wall and the honeycomb structure are fixed in all degrees of freedom except the direction of compression. In the experiment, the loading rate of the roof is 2 mm/min. In the numerical simulation process, mass scale amplification and velocity amplification methods are generally used to simulate in-plane compression behavior in order to save calculation costs. Therefore, an amplification method with a speed of 0.1 m/s is used for the rigid wall in this study.

In quasi-static compression simulations, the accurate selection of the structural mesh size can make the structural simulation analysis more accurate and efficient, thus enabling the structural mesh sensitive analysis. To determine the mesh size, a mesh convergence analysis was carried out, as shown in Figure 3b. Compare the difference between calculation time and calculation SEA, taking into account the simulation accuracy, numerical stability and computational efficiency, ensuring convergence and reducing the amount of operations, the mesh size was determined to be set at 1 mm optimally.

### 2.3. Experimental Setup

**Figure 4:**
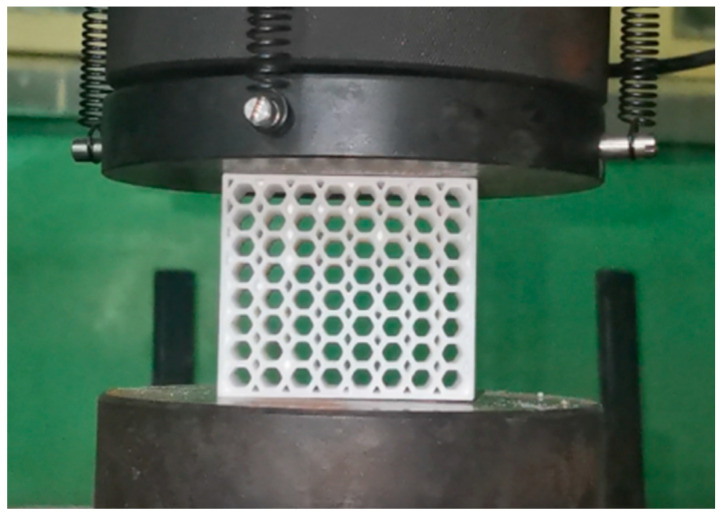
Quasi-static compression experimental equipment.

To verify the accuracy of the finite element model, quasi-static compression tests were carried out on four angular rotating honeycombs using a universal testing machine, as shown in Figure 4. The bottom of the specimen was placed on a rigid base below and the rigid body above compressed the specimen at a rate of 2 mm/min. The variation process of the reaction force with compression displacement was obtained through a force transducer, and the compression test force-displacement curve derived from the electronic universal testing machine system was transformed into a compression test stress-strain curve using Equations (1) and (2) [29] for data processing. At the same time, the deformation process of the specimen was recorded using HD video recording equipment.

(1) $$\sigma =\frac{F}{A}=\frac{F}{LB}$$

(2) $$\varepsilon =\frac{\delta }{H}$$

where σ is the instantaneous compressive stress in MPa; F is the compressive force in N; A is the cross-sectional area within the honeycomb surface in m^2^, ε is the instantaneous strain; δ is the downward displacement of the indenter in m; L, B, and H are the overall width, thickness, and height of the honeycomb, all in mm.

## 3. Results and Discussion

### 3.1. Indicators of Energy Absorption Characteristics

Energy Absorption (EA), Specific Energy Absorption (SEA), Mean Crushing Force (MCF), Initial Peak Crushing Force (IPCF), Crushing Force Efficiency (CFE), and Energy Absorption Efficiency (EAE) were introduced as crashworthiness evaluation indexes, Crush Force Efficciency (CFE), Energy Absorption Efficiency (EAE) [30,31,32,33,34,35,36,37] were used as crashworthiness indicators to evaluate the crashworthiness of rotating honeycomb structures.

(1) Total Absorption Energy (EA)

The total energy absorption of the cellular porous structure undergoing elasto-plastic deformation during crushing is evaluated by the total energy absorption (EA), i.e., the area under the force-displacement curve, calculated as shown below:

(3) $$EA=\int_{0}^{d}F(x)dx$$

where d denotes the crush distance. The greater the total energy absorption EA of a structure at the same displacement, the better the energy absorption capacity.

(2) Specific energy absorption (SEA)

In order to eliminate mass differences between individual models, the specific absorbed energy of a structure is defined in terms of energy absorbed per unit mass and is calculated as shown below:

(4) $$SEA=\frac{EA}{m}$$

where m is the mass of the specimen, it is clear that a higher specific energy absorption indicates a higher energy absorption effectiveness of the structure.

(3) Mean crushing force (MCF)

The mean crush force MCF is a characterisation parameter of the energy absorption of a porous thin-walled structure at unit displacement, the value of which can be obtained from the ratio of the total energy absorption to the crush displacement d. The formula is shown below:

(5) $$MCF=\frac{EA}{d}$$

(4) Initial peak force (IPCF)

The initial peak force of IPCF occurs at the beginning of the collapse of a cellular porous structure and is the first peak impact reaction force generated during the initial phase of the collapse. The initial peak force IPCF makes a small contribution to the energy absorption of the structure and too high an initial peak force IPCF can cause some injury to people in the protective device.

(5) Crushing Force Efficiency (CFE)

It is the ratio of the Mean Crushing Force (F_mean_) to the Maximum Crushing Force (F_max_) and is a key indicator of structural load stability. The larger the crushing force efficiency value, the more stable the deformation, as shown by the following formula:

(6) $$CFE=\frac{F_{mean}}{F_{max}}\times 100\%$$

(6) Energy Absorption Efficiency (EAE)

The energy absorption efficiency η is the ratio of energy absorption to the corresponding stress during quasi-static compression and is calculated as shown below:

(7) $$\eta =\frac{\int_{0}^{\varepsilon }\sigma_{m}d\varepsilon }{\sigma_{m}}$$

(8) $${\frac{d\eta (\varepsilon )}{d\varepsilon }│}_{\varepsilon =\varepsilon d}=0$$

### 3.2. Experimental Results and Model Validation

The deformation patterns of cellular energy-absorbing structures are a key basis for the study of energy absorption properties. It is necessary to explore the deformation patterns or folding types of typical microcells and to clarify the deformation mechanism of the overall structure.

**Figure 5:**
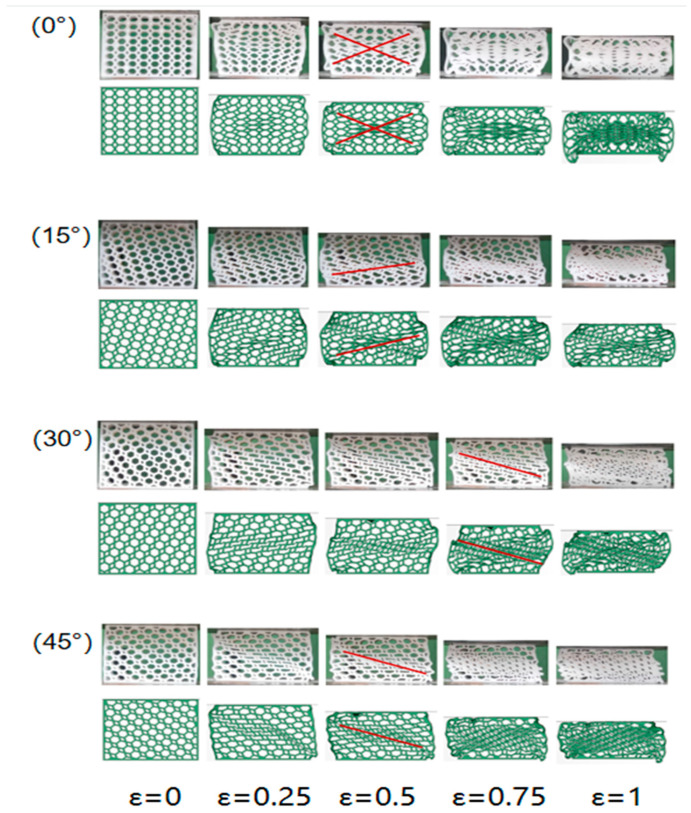
Simulation and experimental deformation diagram.

Typical characteristics of honeycomb structured materials subjected to impact include localisation of deformation and stress enhancement. Figure 5 gives typical deformation patterns for four different angles of the honeycomb structure for compressive strain states.

For the 0° original honeycomb, a “V” shaped deformation zone appears on the upper side of the model first; as the downward pressure increases, an “X” shaped deformation zone appears throughout the model, with the deformation mainly concentrated in the middle part of the model. For the 15° rotating honeycomb, One-piece deformation belt appears first at the lower middle of the model. For the 30° rotating honeycomb, the same One-piece deformation zone appears in the lower central part of the model, but the direction of the zone is opposite to that of the 15° honeycomb. For the 45° rotating honeycomb, the collapse pattern starts diagonally with layer-by-layer compaction, with the gap between the honeycomb cells gradually decreasing to near zero and the compaction area gradually approaching the fixed end.

In the “x” deformation mode, the hexagonal honeycomb expands laterally with the compression. This is because the special structure of the hexagonal honeycomb causes the cell wall to produce a plastic hinge, resulting in the gradual collapse of the hexagonal honeycomb and the formation of an X-shaped shear band. This X-shaped shear band causes cells near the free boundary on both sides of the hexagonal honeycomb to have less capacity than the lateral constraints near the central region, which will squeeze the cells outward, causing lateral expansion. There is no obvious macroscopic deformation at the initial stage of compression. However, the internal cell is slightly bent, and after the stress exceeds the initial peak, the stress is stabilized at the stress value in the plateau period with the increase of strain. The deformation of the overall rotating honeycomb is mainly due to the bending and rotation of the cell wall. Once the inclined wall is crushed, an external bending moment will occur around the node. The deformation zone is gradually expanded layer by layer, which will not cause the transverse contraction and expansion of the structure. Compared with the X-shaped shear zone, the one-shape shear zone has a more stable deformation mode and better self-restraint ability, which is conducive to maintaining stable mechanical properties in practical applications. The deformation of the grouped rotating honeycomb structure is quite different from that of the whole rotating structure. The initial peak force of the grouped rotating honeycomb structure is 1.2–1.45 times that of the whole rotating honeycomb, which indicates that the grouped rotating design has a favorable influence on the crushing load. The structural strength of different components is different, and the load force required for deformation is greatly different. The component that deforms first reaches a dense state before the deformation zone is formed by a large macroscopic deformation in the next component, which is the obvious reason why the impact resistance is better than the whole rotating honeycomb.

### 3.3. Quasi-Static Crushing Response of an Integral Rotating Honeycomb

**Figure 6:**
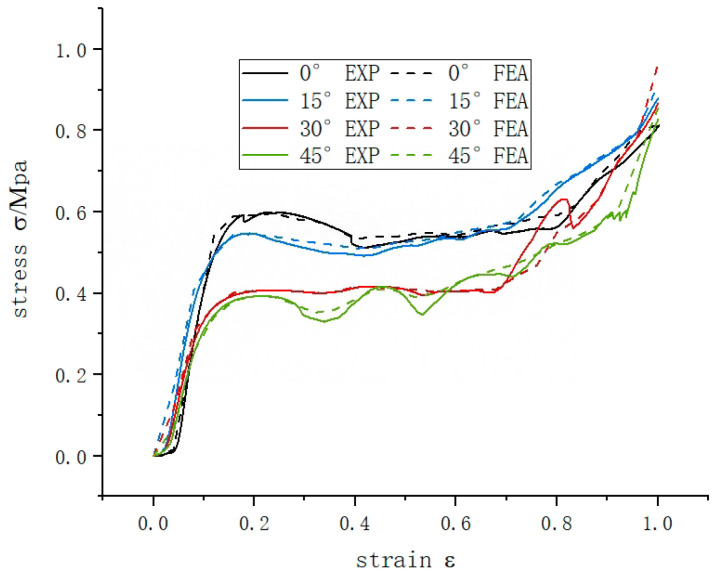
Experimental and simulated stress-strain curves for quasi-static compression of a rotating honeycomb.

The rotating honeycomb has excellent deformation performance as well as good energy absorption characteristics. The force-displacement curves obtained from quasi-static compression experiments, as shown by Figure 6, show that the rotating honeycomb of four angles all have the same three typical stress characteristic phases as the porous structure under forward compression conditions: the linear elastic phase at the beginning of loading (I), the plastic deformation plateau at the middle of loading as the yielding phase reflecting the energy absorption capability of the structure (II) and the crushing densification stage at the end of loading (III). The experimental results are consistent with the simulation results in terms of stress levels and deformation patterns, confirming the accuracy and validity of the finite element simulation.

During the initial stages of loading, the overall experimental model presents the material properties of the nylon material, with the image curve exhibiting linear characteristics. At this stage, the stress curve increases linearly with strain, rising sharply to approximate a straight line and reaching its first peak.

In the middle stage, when the compressive stress of the honeycomb exceeds the yield strength, the load continues to be applied, the deformation increases substantially, the stress tends to level off or even shows a downward trend, theoretically this stage belongs to a performance of yielding, at the same time the porous structure of each layer absorbs energy through its own deformation, the curve shows a smooth and slow rise, so it also belongs to the energy absorption stage, this interval is long, reflecting that the structure has quite good At the same time, during the platform stress phase, as the strain increases, the individual diagonal beams of the honeycomb structure collapse and fail, showing a layer-by-layer folding state, and the stress fluctuates within a certain range, which is determined by the deformation form of the honeycomb.

At the end of loading, the model structure tends to densify and this phase is the damage phase of the model, when the internal cellular elements of the structure are crushed and the stresses increase sharply to their maximum values and the curve increases steeply.

As can be seen from Table 2, the rotating honeycomb structure of equal relative density, the best energy absorption effect of 15° honeycomb is 142 J and 4.02 J/Kg, which is 1.06 and 1.09 times of 0° honeycomb, and the bearing capacity of 15° honeycomb is 1.06 times of 0° honeycomb, meanwhile, the best crushing force efficiency of 15° honeycomb is 65%, which is 2% higher than 0° honeycomb; the stress-strain curve of 15° honeycomb fluctuations were smaller and no frequent fractures occurred. In summary, a structure with better energy absorption should have higher platform stress and lower fluctuations.

The specific absorption energy of honeycomb increased by 8 J over 0 honeycomb, and the average stress increased by 0.3 kN. The deformation behavior and mechanical properties of honeycomb structures depend on their microstructure arrangement or topology, and some unique properties can be obtained by rationally adjusting the microtissue arrangement of materials. The deformation of the honeycomb structure is dominated by bending deformation, which is easy to form a shear band, thus affecting its mechanical properties. The shear band is usually formed along the diagonal direction of the specimen, and strengthening the diagonal of the rotating honeycomb by rotating the diagonal rod can further increase the in-plane stress while creating more plastic hinges, thus increasing the energy absorption capacity of the structure.

**Figure 7:**
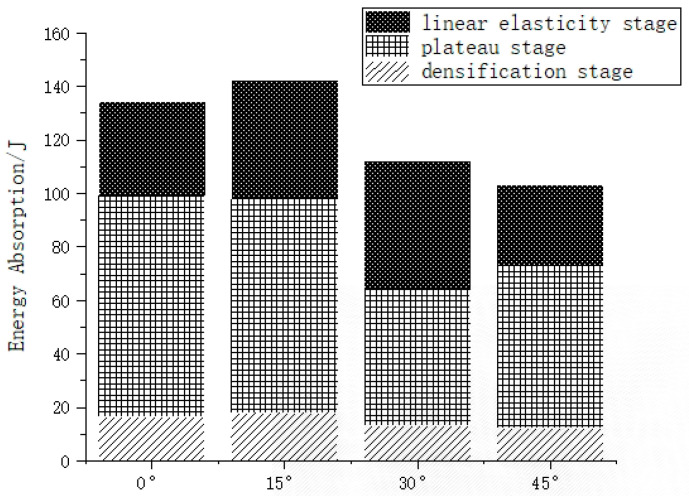
Three-stage energy evolution of a rotating honeycomb structure.

Figure 7 shows that the compression modulus of the 15° honeycomb is the same as that of the 0° honeycomb, and the compression strength is slightly less than that of the 0° honeycomb. The compression strength and compression modulus of the 0° and 15° honeycombs are significantly higher than those of the 30° and 45° honeycombs, due to the changes in the structure of the 30° and 45° honeycombs resulting in the cell walls touching each other during compression, the lateral shrinkage of the structure being more impeded and the compression modulus being lower.

**Figure 8:**
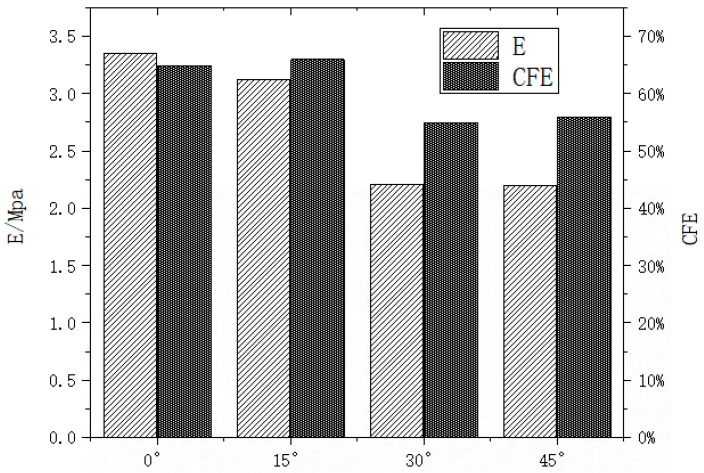
Compression modulus and crush force efficiency in rotating honeycomb quasi-static compression experiments.

**Figure 9:**
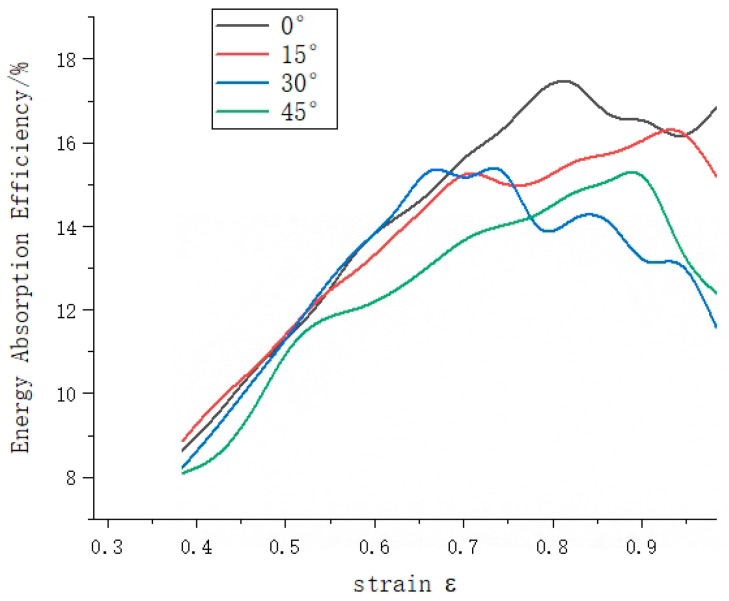
Rotating honeycomb structure energy absorption efficiency curve.

The percentage of energy absorption in the three phases of the compared species of rotating honeycomb, is shown in Figure 8. From Figure 8, it can be seen that the load-bearing capacity of the four rotating honeycombs is more varied in the plateau phase, the energy absorption capacity in the platform plateau phase is the most sensitive to the difference in structure, and the energy absorption capacity in the linear elastic phase is least influenced by the model structure; compared with 30° and 45°, 0° and 15° have better stable plastic crush section and smooth collapse phase; while in the dense phase of 15° honeycomb, the amplitude of the stress curve The 15° honeycomb has a slightly higher amplitude than the 0° honeycomb, so the energy absorption density of the 15° honeycomb eventually exceeds that of the 0° honeycomb. In general, the lattice structure can effectively absorb energy through plastic deformation until it is compacted, and when the energy absorption efficiency reaches its maximum, the structure reaches the densification stage. Figure 9 shows that the energy absorption efficiency of all four types of honeycomb is reflected as first increasing and then decreasing, and the maximum value of 15° honeycomb is smaller than that of 0° honeycomb, but the moment when the maximum value of 15° honeycomb appears is smaller than that of 0° honeycomb. In the compression test, the cell walls of the lattice bend and fracture, providing volume space for further compression of the structure.

### 3.4. Simulation Results of the Combined Design Rotating Honeycomb

**Figure 10:**
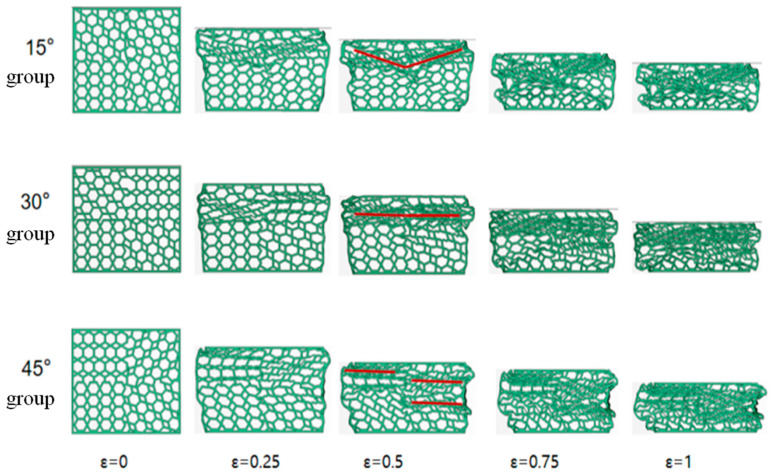
Simulated deformation of a rotating combined honeycomb structure.

Figure 10 compares the deformation patterns of the three rotating combinations of honeycombs. From the deformation diagram, it can be seen that the 15° combined honeycomb shows a small deformation, with a “V” shaped deformation zone at the top, and as the compressive strain increases, the deformation at the impact end gradually increases, and the deformation at the bottom fixed end starts to deform and gradually increases, with an “X” shaped deformation zone. Eventually, the honeycomb structure enters the densification stage and the cellular elements are destructively crushed, the honeycomb structure increases in four sub-regions at close angles, so the shape of the deformation zone is similar to that of the 0° honeycomb; the 30° combined honeycomb has a one-piece deformation zone at the impact end of the honeycomb structure at the beginning of the impact, as the compressive strain increases, more honeycomb cell elements enter the deformation region, the 30° honeycomb is rotated to create vertical supports and each cellular structure is deformed to its limit above and below the vertical rod, resulting in a “cavity” inside; The 45° honeycomb is rotated at a large angle and at a strain of 0.25, the upper honeycomb is deformed while the lower honeycomb is also deformed by tilted rotation, resulting in multiple deformation zones and more instability overall than the 15° and 30° combinations.

**Figure 11:**
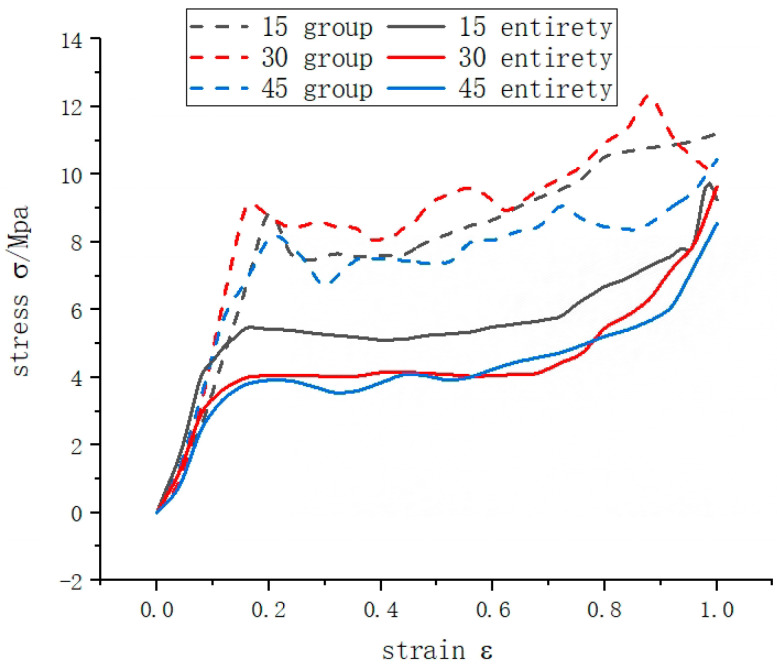
Combined rotating honeycomb quasi-static compression simulation stress-strain curve.

Compared to the integral rotating structure, the stress curve of the grouped rotating structure (as shown in Figure 11) has no obvious plateau period, when impacted, the upper structure deforms before the lower structure, when compression gradually increases, the lower structure still has the ability to deform and absorb energy, the stress-strain curve continues to increase upwards, there is no horizontal plateau period; the slope of the curve of the 15° honeycomb elastic deformation stage 1 is higher than that of stage 2, while The 30° and 45° honeycombs pass through a buffer zone in the second stage and the slope of the curve is close to that of the initial stage. The combined design can compensate for the weakening of the energy absorption capacity of the overall deformation of the structure at a later stage.

The 30° honeycomb has a higher first stage curve than the 15° and 45° combinations, the initial peak force IPCF is the highest of the three (as shown in Table 3), the average crushing force, total energy absorption and specific energy absorption of the 30° combination are better than the other two combinations, but the initial peak force of the 15° combination is lower than the 0° and 15° compared to the overall honeycomb, and the energy absorption level of the 30° combination is higher than the overall 15° honeycomb, suggesting that the combination honeycomb can reduce the peak force of the structure while increasing the level of energy absorption.

Compared with the typical third-order process of overall honeycomb, the combined rotating honeycomb consists mainly of two stages, linear elastic stage and fluctuating plateau, which introduces higher platform stress, and the results show that the combined rotating honeycomb structure has excellent energy absorption capacity. The deformation range of the combined honeycomb structure is wider, which can more effectively bear the surface compression load, and more cell walls can produce more plastic hinges, thus generating more energy dissipation in the plastic deformation stage; the combined rotation structure can improve the modulus and energy absorption capacity of the material, for application scenarios with auxiliary and bearing performance requirements; its characteristics can be further adjusted by adjusting the Angle value of the combined structure. It should be noted that this paper focuses on the quasi-static loading case, considering the large impact velocity range of protective structures in practical applications, the results of the rate on the mechanical properties of the rotating honeycomb are discussed, which requires further study.

## 4. Conclusions

In this paper, the mechanical behaviour, in-plane impact response and energy absorption mechanism of the integral rotating honeycomb structure are investigated through quasi-static compression tests and finite element simulations by comparing 0°, 15°, 30°, 45° rotating honeycomb and 15°, 30°, 45° combined honeycomb, and based on this, initial peak force, platform stress, energy absorption and specific energy absorption are used to show the influence of compression performance in the plane.The comparative analysis of The deformation patterns and energy absorption characteristics of the combined honeycomb structures were investigated and the main conclusions were as follows:

(1) Deformation patterns: 0° honeycomb has a typical “X” shaped deformation zone, 15°, 30° and 45° are more obvious zigzag deformation zones, all are more uniform in-plane compression deformation patterns; combined rotating honeycomb has obvious layered deformation characteristics, the upper layer deforms first, the lower layer deforms later, and the local deformation zone is obvious.
(2) In terms of platform stress: both the 15° honeycomb and the 0° honeycomb outperform the 30° honeycomb and the 45° honeycomb, and the 15° honeycomb is higher than the 0° honeycomb in terms of average crush force and crush force efficiency, but in the combined rotary honeycomb, the 15° combination is smaller than the 30° and 45° combinations in terms of overall stress-strain curve and average crush force. Compared with the integral honeycomb, the combined honeycomb has higher initial peak force and platform stress, which plays an important role in the energy absorption of the structure.
(3) Energy absorption: The energy absorption capacity of 0 and 15 is significantly higher than that of 30 and 45. The total energy absorption of the 15, 30 and 45 honeycomb was 194 KJ, 213 KJ and 187 KJ, which are 35%, 73% and 71% higher than the overall honeycomb, respectively, 15, 7.76 kN, 8.52 kN and 7.36 kN, respectively, and the 30 honeycomb was better in the combined cells.

It can be seen that, compared to 0° honeycomb, the different pressure-bearing directions of the rotating honeycomb cell walls lead to significant changes in the compressive deformation pattern and energy absorption properties of the honeycomb material, and the rotating design of the cell walls can enhance the impact resistance of the honeycomb. The overall rotational and combined rotational design strategies proposed in this study provide a new way to design lattice structures with controllable deformation mechanisms and adjustable mechanical properties.

## Figures and Tables

**Table 1:** Rotary honeycomb size, parameter quality table.

Honeycomb Angle	Length (mm)	Width (mm)	Height (mm)	Mass (g)
0°	60	30	50	36.523
15°	60	30	50	35.295
30°	60	30	50	34.085
45°	60	30	50	35.595
15° combination	60	30	50	35.485
30° combination	60	30	50	35.023
45° combination	60	30	50	35.62

**Table 2:** Analysis of energy absorption results.

		0°	15°	30°	45°
IPCF (kN)	Experiment	5.9	5.5	4.1	3.9
MCF (kN)	Experiment	5.4	5.7	4.8	4.5
EA (J)	Experiment	134	142	120	113
	Simulation	136	143	118	115
	Error/%	−2.2	−0.7	1.67	−1.7
SEA (J/g)	Experiment	3.7	4	3.5	3
	Simulation	3.6	3.95	3.4	3.1
	Error/%	−2.7	−1.25	-2.8	3.3

**Table 3:** Analysis of combined rotary honeycomb energy absorption results.

Honeycomb Angle	IPCF (kN)	MCF (kN)	EA (J)	SEA (J/g)
15° combination	9.3	7.76	194	5.4
30° combination	9.2	8.52	213	6.08
45° combination	8.2	7.36	184	5.13

## Data Availability

This study did not report any data.
